# Supplementary material for: Tularemia Goes West: Epidemiology of an Emerging Infection in Austria
Source: Microorganisms. 2020 Oct 16;8(10):1597. doi: 10.3390/microorganisms8101597 (PMC7602993; doi:10.3390/microorganisms8101597)
Supplement: Supplementary file 1 [file microorganisms-08-01597-s001.pdf]

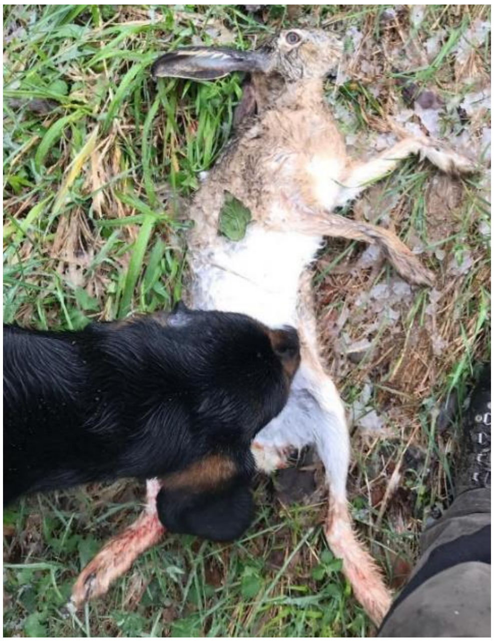

(a)

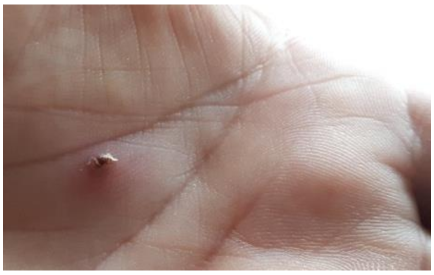

(b)

Figure S1 a and b: Ulceroglandular infection with *F. tularensis* originating from an infected brown hare **(a)** and ulcer in the palm of patient 1 **(b)** acquired in Tyrol in 2018.
